# Caffeine content in filter coffee brews as a function of degree of roast and extraction yield

**DOI:** 10.1038/s41598-024-80385-3

**Published:** 2024-11-25

**Authors:** Zachary R. Lindsey, Joshua R. Williams, James S. Burgess, Nathan T. Moore, Pierce M. Splichal

**Affiliations:** 1 Department of Physics, Berry College, Rome, GA USA; 2 Department of Chemistry, Drexel University, Philadelphia, PA USA; 3 Department of Chemistry, Berry College, Rome, GA USA

**Keywords:** Chemistry, Materials science

## Abstract

The effect of degree of roast on resulting caffeine content in brewed coffee has been extensively researched, but conflicting methodologies and results have muddled development of a general conclusion. In this study, 30 unique combinations of green coffee variety, degree of roast, and brew time were investigated regarding extraction yield and caffeine content. An AeroPress brewer was used to prepare brew samples using a 15:1 mass ratio of brew water to ground coffee. Refractometry and HPLC were respectively used to measure extraction yield and caffeine content of brewed samples. Scanning electron microscopy was used to measure porosity of roasted seeds and showed increasing porosity with the degree of roast. Extraction yields generally decreased for roast batches with roasting mass losses greater than $\sim$12–14%, and caffeine concentrations in 10-min brews decreased for roast batches with drop temperatures greater than $\sim$400–420 $^{\circ }$F. Under identical brewing conditions, caffeine concentrations in brewed samples were generally lower for dark roasts than light and medium roasts. However, at identical extraction yields, dark roasts generally exhibited higher caffeine concentrations than lighter roasted coffees. It is likely that the volatilization or decomposition of soluble compounds and increased porosity due to roasting act as competing mechanisms that determine compound concentrations in resulting brews.

**Supplementary Information:**

The online version contains supplementary material available at 10.1038/s41598-024-80385-3.

## Introduction

The concentration of caffeine in brewed coffee is a topic of public interest based on the global popularity and health implications associated with consumption of the beverage^1^. Previous studies have investigated possible links between consumption of coffee/caffeine and several health benefits, including lower risks of liver disease^2^, heart disease^3^, Parkinson’s disease^4^, Alzheimer’s disease^5^, type 2 diabetes^6^, depression^7^, and some forms of cancer^8^. The effect of the degree of roast on the resulting caffeine content in the cup has been extensively researched in more than 20 studies performed over the past few decades. Among these, the variety and disagreement among published results is staggering. While the most commonly reported result in the literature is that caffeine content is unaffected by roast degree^9–16^, there are multiple studies that report a roast degree dependence resulting in higher caffeine concentrations for lighter roasts^17–22^, medium roasts^23–27^, and for darker roasts^28–31^. One possible reason for the breadth of conflicting results is the difficulty of isolating the effect of roast degree by minimizing contributions from other experimental parameters. Since changes in caffeine concentration related to degree of roast are relatively minor, scrupulous control over all other stages of the experimental process is pivotal to avoid effects from confounding variables. In this study, each step of the experimental process was meticulously documented and quantified, including green coffee sourcing, roasting, degassing, grinding, sieving, brewing, and characterization.

There is currently no standard method by which the degree of roast is universally and precisely quantified. In fact, there are many physical quantities that can serve to partially describe the degree of roast, including (but not limited to) color, density, weight/mass loss, total roast time, roast time after the onset of first crack, and initial (“charge”) and terminal (“drop”) roast temperatures of the roast batch^32–34^. Variations in the determination of degree of roast among previous studies is another factor that likely contributes to the vast disparity among results. In this study, all of the parameters mentioned above were measured for each roast batch to provide a robust description of the degree of roast. After roasting, variations in particle size distributions achieved through grinding can play a significant role in extraction kinetics that occur during the subsequent brewing step^35,36^. In order to minimize the effects of varying grind distributions, a sieve step was implemented to further isolate the effect of degree of roast on caffeine extraction.

In addition to caffeine content, the effect of degree of roast on extraction yield of resulting coffee brews was also investigated. Few previous studies have researched the role of roast degree on extraction, and there are discrepancies among results. Most of these studies only quantified changes in “brew strength” via measurements of total dissolved solids (TDS), and conflicting trends of increasing^10,37^ and decreasing^38^ TDS values with increasing degree of roast were reported. Also, one study reported indistinguishable equilibrium TDS values in full immersion coffee brews as the degree of roast was varied^39^. It is worth mentioning that each of the brew methods employed for these studies showed slight variations and likely contributed to inconsistencies among results. The chemical composition of brew water also influences compound extraction in coffee brewing. The effects of water chemistry on resulting extraction rates and compositions in brewed coffee have been investigated both experimentally^40–42^ and computationally^43^ in previous studies. In this work, water composition and brew method were carefully and consistently controlled for all brewed samples in order to minimize undesired contributions from any variables other than those being investigated.

While different grind size distributions affect extraction rates and yields due to changes in particle surface area to volume ratios and diffusion path lengths^44^, the microstructure of the roasted coffee grains also plays a significant role in extraction dynamics^45^. As pressure builds up within the coffee seeds during roasting, their volume and porosity increase. When internal pressure reaches a critical point, the structure of the coffee seed matrix breaks down, primarily releasing trapped water vapor and $\hbox {CO}_2$. This study investigated the evolution of porosity as the degree of roast was changed and compared porosity data with caffeine concentration and extraction yield measurements. This research represents the first instance in the literature of a quantitative comparison between caffeine content, extraction yield, and porosity as a function of the degree of roast.

## Experimental details

### Green coffee

All green coffee used in this work was obtained from Royal Coffee (Emeryville, CA). The study was performed using a natural process Ethiopian green coffee (Organic Chelbesa Raised Bed—Crown Jewel CJO1503) and a fully washed process Ethiopian green coffee (Ethiopia Yirgacheffe Grade Zero Washed Adame Kebele—28183). Also referred to as “dry-processed”, natural process coffees are produced by allowing the coffee cherries to dry and ferment in the sun over the span of several days before the husks and surrounding fruit are mechanically removed. Conversely, washed process coffees (sometimes referred to as “wet-processed”) have the outer fruit removed prior to fermentation, and water is used to assist in removing the remaining mucilage layer before grading and sorting^46^. The Ethiopian natural green coffee was grown at an elevation of 1900–2200 m above sea level, and moisture content and freely settled density measurements were reported by Royal Coffee to be 10.5% and 0.707 g/mL (respectively). The Ethiopian washed green coffee was grown at an elevation of 1950–2100 m above sea level, and moisture content and freely settled density measurements were reported by Royal Coffee to be 9.6% and 0.692 g/mL (respectively).

The different green coffees used in this study were not selected for specific investigation of the impact of processing style on resulting extracted compounds. Instead, two coffees exhibiting differing behaviors under similar roasting conditions were selected in order to observe variations in degree of roast and extraction characteristics.

**Fig. 1:**
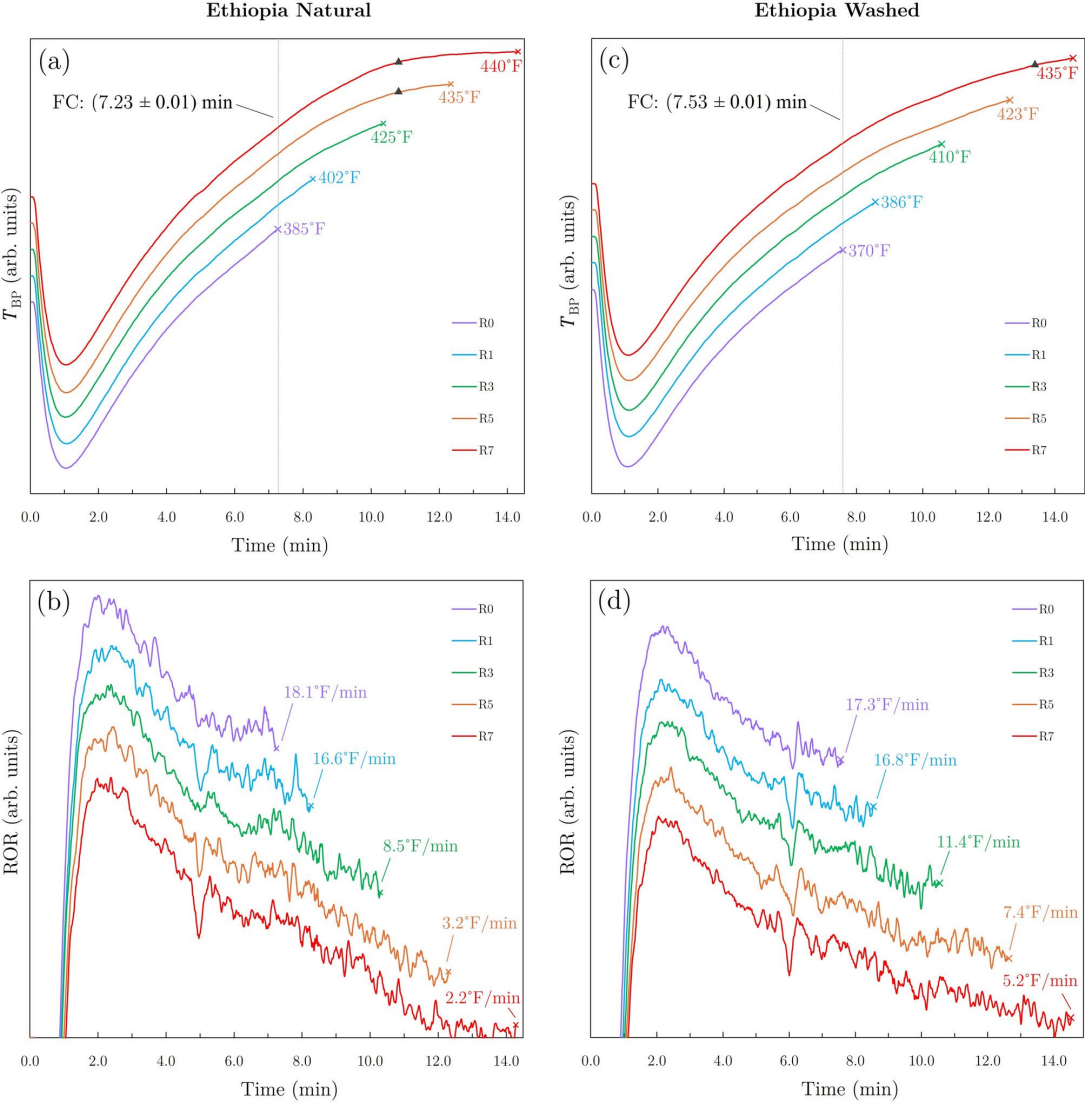
Plots of bean probe temperature ($\hbox {T}_{BP}$) vs roast time (top) and rate of rise (ROR) vs roast time (bottom) for the natural (**a**,**b**) and washed (**c**,**d**) Ethiopian coffee roast batches (plots are vertically offset for ease of comparison). Drop temperatures and final values of ROR are indicated for each degree of roast. FC and SC events are respectively indicated by the dashed vertical lines and triangular markers.

### Roasting method

All roasts were performed using an Aillio Bullet v2 electric drum roaster with 500 g batches of green coffee. Measured masses of green and roasted coffee were obtained via an Adam Equipment ADP-800L balance, and all reported bean temperatures were measured using an NTC thermistor probe located inside the roaster drum. The desired degree of roast for each batch was achieved by implementing a common recipe and dropping the roasted coffee at varying times after the onset of first crack (FC). Roast batches were identified using the format R*N*, where *N* corresponds to the roast time (in min) after the onset of FC. Roast recipes were designed for each green coffee using RoasTime 4 software so that the first derivative of $T_{BP}$ vs roast time (also known as the rate of rise) exhibited a consistently decreasing trend for the entirety of the darkest (R7) roast. Once R7 roast recipes were finalized for each green coffee, the remaining degrees of roast were achieved by simply terminating the roasts at earlier times.

For each of the green coffees investigated in this study, 5 different degrees of roast were achieved using the described approach (R0, R1, R3, R5, R7). Roasting curves for the natural and washed Ethiopian coffees are shown in Fig. 1. All roast batches for the natural and washed Ethiopian coffees were initiated at respective charge temperatures of $330\,^{\circ }\hbox {F}$ and $340\,^{\circ }\hbox {F}$. The FC event was determined by recording the time at which 3 or more audible cracking noises were observed within a duration of 3 seconds. In preliminary roasts performed to roughly assess FC event timing for each green coffee, FC times were repeatedly observed and recorded in multiple trial roasts before performing the final roasts used for sample preparation. A second crack (SC) event was observed for the R5 and R7 natural and R7 washed Ethiopian roast batches. After each batch was dropped out of the roasting drum, the roasted coffee was air-cooled and stirred for 2–3 min until the coffee was near room temperature. The roasted coffee was then packed and sealed in aluminum-lined bags equipped with one-way valves and stored in a climate-controlled lab. The subsequent grinding, sieving, and brewing steps were performed after a 10-day degassing period (relative to the roast date) for each roast batch.

### Grind size control

**Fig. 2:**
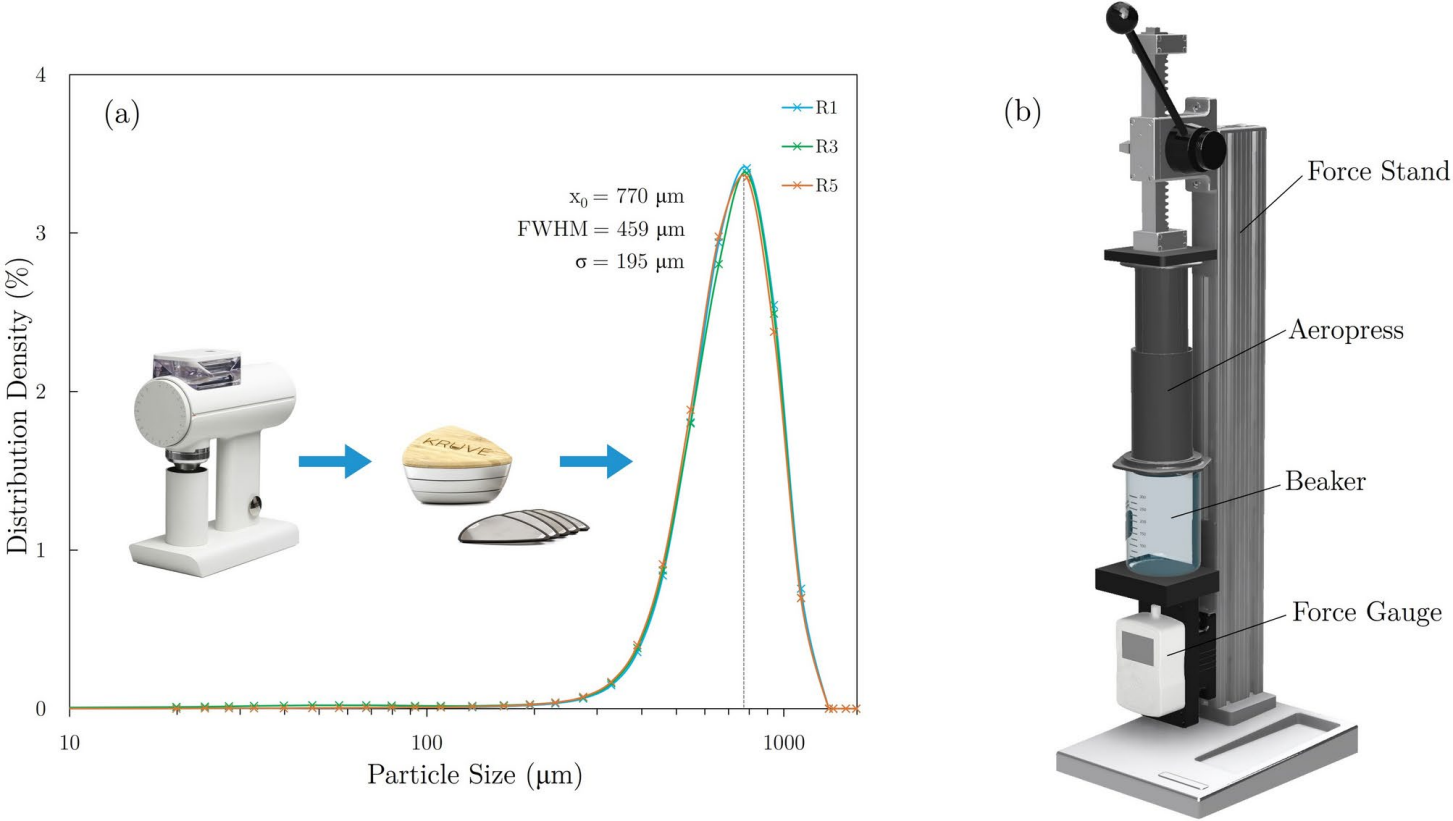
(**a**) Volume distribution density vs coffee particle size (resulting from grind/sieve process) obtained by laser diffraction of 3 different roast batches of the natural Ethiopian coffee, and (**b**) brewing apparatus consisting of an AeroPress atop a glass beaker housed within a force stand equipped with a manual force application lever system and digital force gauge.

Following the 10-day degassing period, each roast batch was ground using a Timemore Sculptor 078 grinder ($$\sim 1000$$ rpm; burr spacing “5”) and then sieved for 10 min at ($$200\pm 20$$) rpm using a Kruve Sifter Plus equipped with $$700\, \upmu \hbox {m}$$ and $$900 \,\upmu \hbox {m}$$ mesh sieves. Particle size analysis was performed using a Sympatec HELOS-KR laser diffraction instrument, and volume-specific particle size distributions were obtained via Fraunhofer Enhanced Evaluation mode. The sieve step was implemented after various roast batches showed highly variable features within the grind distributions, including inconsistencies related to a broad secondary peak composed of coffee particles $$<200\mu$$m in diameter (often referred to as “fines”). After implementing the coupled grind/sieve step, particle size distributions obtained for samples from various degrees of roast and coffee type exhibited consistent unimodal distributions (as shown in Fig. 2a) with mean particle size ($$x_0)$$, full width at half maximum (FWHM), and standard deviation ($$\sigma$$) respectively determined to be 770 $$\upmu$$m, 459 $$\upmu$$m, and 195 $$\upmu$$m. The grind/seive step was performed on the day of brewing for each roast batch.

### Degree of roast characterization

In addition to temperature and time data obtained during roasting, each roast batch was characterized in terms of color (via reflectance spectroscopy), density, and percent mass decrease (see Fig. S1 in Supplementary Materials). Color measurements were performed using a Javalytics Degree of Roast Analyzer (JAV-RDA-U) and reported using the Agtron gourmet scale. Color measurements were obtained from ground and whole bean samples at the end of the 10-day degassing period. Reported values represent the average of 4 independent measurements, each taken after rotating the sample dish $\sim 90^{\circ }$. Freely settled density measurements were also obtained using ground/sieved samples of each roast batch following the 10-day degassing period. Mass decrease measurements serve as a means to directly observe mass loss during the roasting process (when obtained immediately after the roast batch is dropped and cooled). Continued mass measurements obtained over an extended period after each roast enable monitoring of additional losses and degassing rates attributed to residual $\hbox {CO}_2$ release. While post-FC roast time was the primary quantity used to control the degree of roast throughout the study, measurements of drop temperature, total roast time, color, density, and mass decrease provide additional support and transparency for degree of roast designations.

Commercial roasters often document relative percentages of roast time spent in 3 different phases of the roasting process. These include a drying phase, a middle phase, and a post-FC “development” phase. The drying phase begins when the green coffee is introduced into the hot roaster and ends when the moisture content of the coffee has been sufficiently depleted so that select chemical reactions can begin. The end of this phase is typically informed by sensory cues such as color change of the roasting seeds from green to yellow and production of a hay-like aroma^47^. In this study, the end of the drying phase for all roasts performed was defined as the time at which the bean probe temperature reached 300 $^{\circ }$F. The middle phase (also referred to as the “browning”, “caramelization”, or “Maillard” phase) begins after the drying phase and extends until the onset of FC. A variety of chemical reactions, such as pyrolysis, Strecker degredation, and Maillard reactions, begin during this phase and significantly contribute to flavor and texture development in resulting coffee brews^47–50^.

The middle phase ends at the onset of FC, which initiates the final phase of the roast. While this portion of the roast is often referred to as the development phase, the term “development” in coffee roasting is often used to describe the process of decreasing the temperature gradient between the inner core and outer surface of roasting coffee seeds. While this process is initiated soon after the green coffee enters the roaster and continues for the duration of the roast^34^, the roasting period between the onset of FC and the end of the roast is referred to as post-FC development time throughout this study. Absolute and relative roasting times spent in each phase for all roasts performed in this study are documented in the Supplementary Materials.

### Brew method

A priority of this work was to implement a brew method that would be relevant to the general population of coffee consumers and also facilitate repeatable control of brew parameters. Thus, an AeroPress brewer outfitted with a disposable paper filter was used to prepare brews (via the inverted method) throughout the study. Brew water was prepared using appropriate masses of $\hbox {MgSO}_4$, $\hbox {NaHCO}_3$, and deionized (DI) $\hbox {H}_2\hbox {O}$ (resistivity $\ge$ $18.2\hbox { M}\Omega \hbox { cm}$) to achieve a nominal carbonate hardness of 40 ppm $\hbox {CaCO}_3$^51^. Brew water was prepared in 1 L batches, and carbonate hardness of each batch was measured with a Hannah Instruments HI772 Handheld Colorimeter. The average measured value of carbonate hardness across all brew water batches was 39 ± 2 ppm $\hbox {CaCO}_3$.

A nominal 15:1 ratio of prepared brew water (150 g) to ground coffee (10 g) was used for all brews. After the ground coffee was poured into the inverted AeroPress and gently agitated to level the coffee bed, a digital kettle outfitted with a T-type thermocouple probe was used to heat the brew water to (212 ± 1) $^{\circ }$F. A timer was initiated once the heated brew water made initial contact with the coffee grinds inside the AeroPress. Once the AeroPress was filled with the target mass of brew water, it was quickly capped and gently swirled 5 times to achieve consistent mixing of brew water and grinds. Once the target brew time was reached, the AeroPress was quickly rotated and placed on top of a glass beaker. The brewer/beaker stack was then placed onto a force stand (as shown in Fig. 2b) where a lever was engaged to compress the coffee bed with the AeroPress plunger.

The force stand was used to achieve consistent plunge forces ($\sim$1000 N) and plunge times (15–20 s) before immediately decanting the brewed coffee into 30 mL glass sample vials. In preliminary brew trials, an AeroPress brewer was fractured under an applied load of $\sim$1100 N. Thus, the target plunge force of 1000 N was selected in order to achieve maximum removal of brewed liquid from the coffee bed without damaging the brew setup. Different brew times of 1, 2, and 10 min were selected to controllably vary the extraction yield of resulting brews. Each unique brew combination of the various roast designations (R0, R1, R3, R5, R7) and brew times (1, 2, 10 min) was performed in triplicate.

### Brew characterization

A handheld refractometer (VST Lab Coffee III) was used to measure total dissolved solids (TDS), and extraction yield (EY) was determined for all brewed samples using the equation

1 $$\begin{aligned} EY = \frac{C_B M_B}{M_D}, \end{aligned}$$

where $C_B$ is the brew concentration (TDS), $M_B$ is the brew mass, and $M_D$ is the mass of the dose of coffee grinds. For TDS measurements, all necessary materials and samples were stored in a climate controlled lab in order to encourage temperature stability during data collection. Before each individual sample was measured, DI $\hbox {H}_2$O was used to zero the refractometer, and the temperature of the water inside the sample well was measured. Each sample was gently inverted 10 times before drawing 2.5 mL into a syringe. Once filled with 2.5 mL, a 13 mm nylon syringe filter (0.22 $\upmu$m) was attached via Luer Lock connection, and the sample was forced through the filter into a glass beaker. A plastic pipette was used to transfer 8–10 drops of the filtered sample onto the refractometer sample well where the TDS measurement was taken 3 times over a span of $\sim$1 min. The temperature of the sample (within the sample well) was obtained during each measurement and compared with the recorded temperature of the DI $\hbox {H}_2$O used to zero the device, and the mean temperature difference between these values for all TDS measurements was $\Delta$T$=$(0.12 ± 0.05) $^\circ$C.

High-performance liquid chromatography was used to measure caffeine concentrations for all samples. All HPLC measurements were performed on a Shimadzu Prominence-i LC-2030C 3D Plus HPLC instrument equipped with a diode array detector which allows for collection of 3D data. An ACE Excel 5 C-18 column (150 mm $\times$ 4.6 mm, 5 $\upmu$m) was used with a mobile phase consisting of methanol, acetonitrile, 1M acetic acid, and ultrapure water. The methanol and acetonitrile used were spectrophotometric grade, and the acetic acid was prepared using glacial acetic acid and ultrapure water. The column and detector flow cell were both maintained at 40 $^{\circ }$C. A flow rate of 1.000 mL/min was used, with a run time of 9.5 min for each sample. The acetonitrile and 1M acetic acid components of the mobile phase were held constant (3% acetonitrile, 10% 1M acetic acid) through each run, with methanol and ultrapure water varying. The methanol concentration started at 19% for the first minute, dropped linearly to 10% at 4.5 min, then increased linearly back to 19% at 5.5 min, followed by a linear increase in methanol content to 55% at 6 min with a 1 min hold to push all other compounds off of the column. The methanol content was then reduced linearly to 19% and held for the final minute of each run to re-equilibrate the column in preparation for the next injection. Ultrapure water made up the remaining component of the mobile phase. Three-dimensional absorbance data was collected, from 210 to 700 nm, for the entirety of each sample run. Chromatograms were extracted at 272 nm to provide optimum signal-to-noise ratio for the caffeine peak. Determination of caffeine concentration was performed using 5 external standards with caffeine concentrations ranging from 100–300 ppm. New calibration data was collected with each batch of unknown samples, and each calibration curve was highly linear ($\hbox {R}^2$ $\ge$ 0.9995). The caffeine concentration variation for replicate injections with this instrument and method was <0.5% RSD.

In addition to caffeine, relative concentrations of select chlorogenic acids (CGAs) were measured to observe changes attributed to the degree of roast and to serve as a control reference. Chromatograms were extracted at 333 nm in order to obtain optimum signal-to-noise ratio for the CGA absorbance peaks in the chromatograms. Since no external standards were purchased for these acids, integration of HPLC peaks corresponding to 4-caffeoylquinic (4-CQA) and 5-caffeoylquinic (5-CQA) acids provided relative concentrations of respective CGA isomers. The correct assignment of the CQA isomers to the chromatographic peaks was confirmed by tandem high-resolution mass spectrometry^52^. CQA isomer identification was performed via tandem mass spectrometry using a Thermo Q-Exactive orbitrap instrument coupled to a Vanquish UPLC. The gradient used for chromatographic separation was the same as that used for the caffeine quantification via HPLC, although a smaller dimensioned C18 column was used at a lower flow rate. All mass spectrometry data (MS and MS/MS) was collected in positive-polarity electrospray ionization mode.

### Determination of porosity

The effect of roast degree on seed porosity was investigated by implementing an elliptical mapping method to SEM images obtained for coffee seeds of each roast batch of the washed Ethiopian coffee. Before imaging, seeds were cleaved along a common plane to expose a central cross-section. Coffee seeds were not sputtered or coated prior to imaging, and all seeds were imaged at the same working distance (26.3 mm) using a low accelerating voltage (1.5 kV) to minimize charging. After cleaving, the seeds were imaged at several points located in various regions across the cleaved surface. To account for variation among seeds, porosity measurements were performed in duplicate (2 seeds per roast batch). Since pore gradients were observed relative to the image location on the cleaved surface, 3 images were obtained at various locations across each individual seed (6 total images per roast batch).

**Fig. 3:**
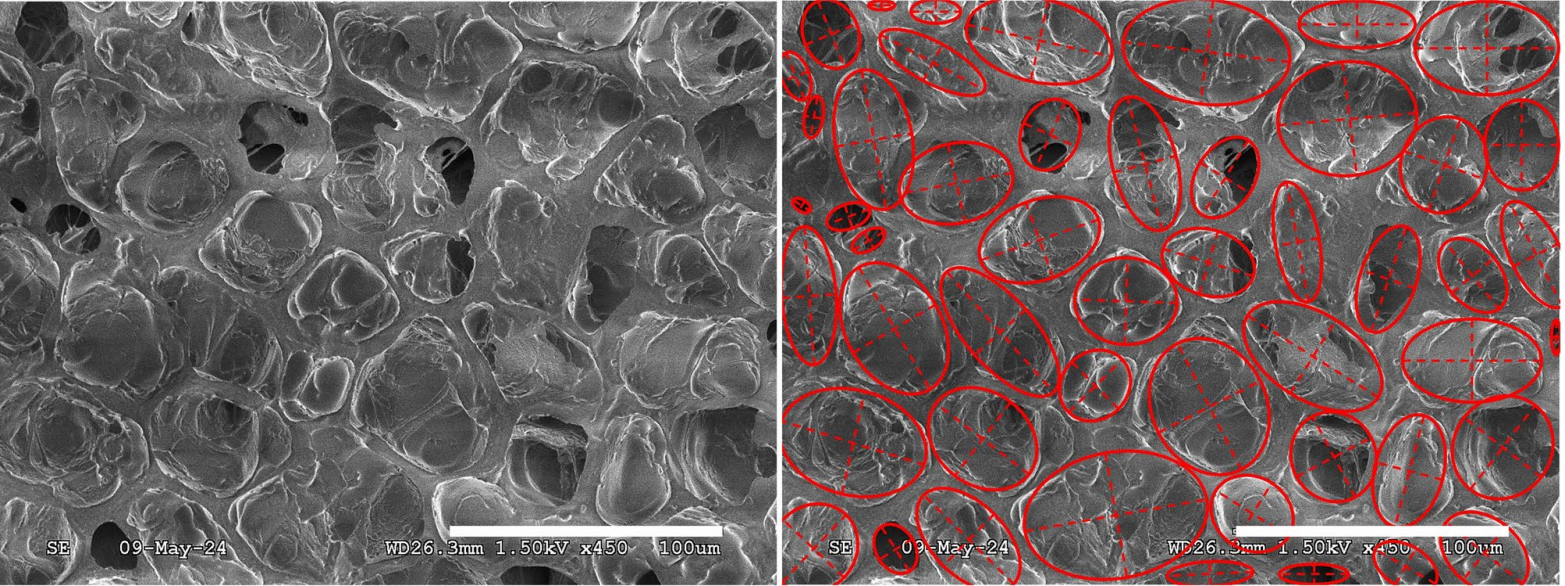
SEM image of roasted coffee seed (left) and overlaid ellipses mapped to pores (right) for determination of fractional porosity (scale bar = 100 $\upmu$m).

For each image, an ellipse was mapped onto each individual pore by assigning major and minor axes lengths to pore dimensions as shown in Fig. 3. Once ellipses were assigned to each pore, the combined area of all mapped ellipses ($S_E$) was calculated and compared to the total area of each image ($S_T$) to determine the fractional porosity ($\%P$), given as

2 $$\begin{aligned} \%P = \frac{S_E}{S_T} \times 100. \end{aligned}$$

In addition, pore density (total number of pores per area) and average pore area were determined for each image.

## Results

In this study, a total of 2 green coffee varieties were roasted to 5 degrees of roast, and brew times of 1, 2, and 10 min were implemented for each roast batch. Each of 30 unique combinations of green coffee, degree of roast, and brew time were performed in triplicate, resulting in a total of 90 brewed samples for analysis.

### Degree of roast quantification

As the post-FC roast time was increased (R0$\rightarrow$R7), the degree of roast progressed from “lighter” to “darker” roasts (using common consumer terminology for degree of roast). While similar roast recipes were implemented for roasts of the natural and washed Ethiopian green coffees, measurable differences in degree of roast characteristics for R0, R1, R3, R5, and R7 roast batches were observed due to variations in composition and size of the different green coffee types. Thus, color, density, and percent mass decrease measurements were used to further quantify the degree of roast of each individual roast batch (all degree of roast data can be found in Supplementary Materials).

Figure S2 (in Supplementary Materials) clearly shows the disparity observed in roasting losses of the natural and washed Ethiopian coffee batches. In general, the washed coffee batches resulted in percent mass decreases that were consistently 2–3% lower than natural coffee roast batches with the same post-FC roast time designation. While the roast recipes and total roast times were similar, drop temperatures varied between the natural and washed coffees for common roast designations. Along with consistently higher density and color measurements and lower drop temperatures, these results suggest that the washed Ethiopia coffee batches achieved systematically lighter roast levels relative to the natural Ethiopian coffee batches. In addition, the R0 and R1 washed Ethiopian batches would likely be described as “underdeveloped” by the specialty coffee community as indicated by the roasting mass losses of 8.9% and 11.6% (respectively).

**Fig. 4:**
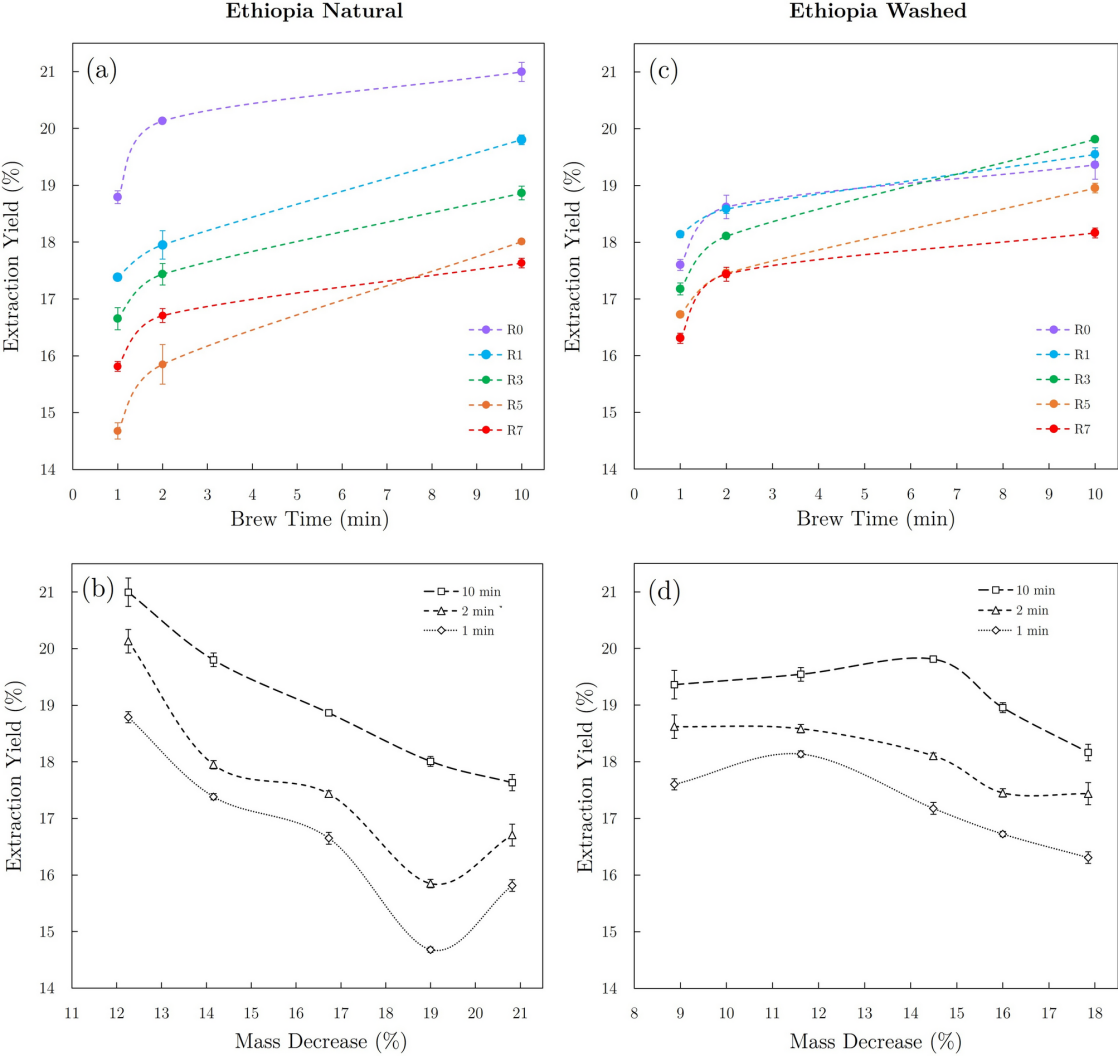
Plots of extraction yield vs brew time (top) and extraction yield vs mass decrease (bottom) for natural (**a**,**b**) and washed (**c**,**d**) Ethiopian coffees. Spline fits were used to clearly differentiate data sets for different degrees of roast or brew time.

### Extraction yield

As shown in Fig. 4, extraction yield measurements showed a general trend of lower extraction yields for darker roasts (R5, R7) than light and medium roasts (R0, R1, R3). The natural Ethiopian roast batches resulted in a wider distribution of extraction yields ($\sim$15−21%) when compared to washed Ethiopian coffee brews ($\sim$16−20%), which seems to have resulted in wider distributions of caffeine concentrations in natural Ethiopian brews. The role of extraction yield on caffeine concentration will be discussed in the next section.

While 1-min and 2-min brew sets showed similar general extraction trends as the 10-min brew sets, the 10-min brew sets resulted in the highest extraction yields among brew times. Extraction yield measurements for 10-min brews of the natural Ethiopian coffee displayed a maximum for the lightest roast batch (R0) and a minimum for the darkest roast batch (R7), exhibiting a consistent decrease in extraction yield as the degree of roast increased (see Fig. 4b). In comparison, the 10-min brew set of the washed Ethiopian coffee showed similar results, as the darkest roast batch (R7) resulted in the lowest average extraction yield. However, extraction yield was highest in R3 brews and showed slight decreases for the lighter roasted R0 and R1 batches. This decrease in extraction yield for the lightest roasts of the washed Ethiopian coffee is likely due to lower porosities inhibiting extraction kinetics within roasted seeds in the underdeveloped R0 and R1 batches.

**Fig. 5:**
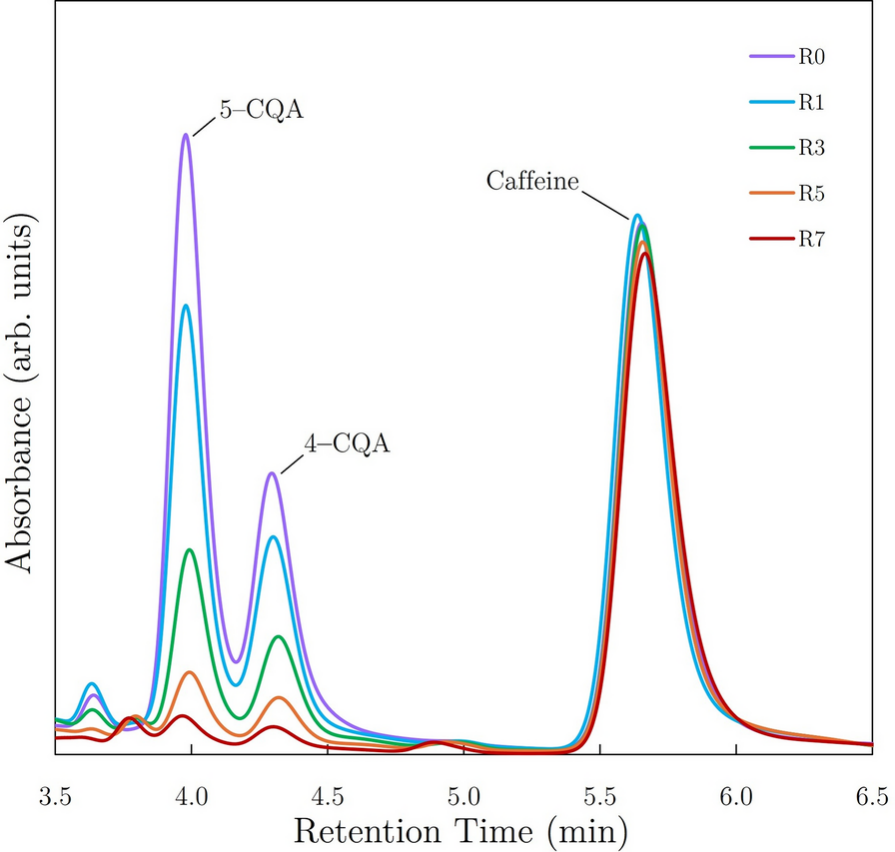
HPLC data showing changes in CGA and caffeine content due to degree of roast for 10-min brews of the Ethiopian natural coffee.

### HPLC characterization

#### Caffeine content

**Fig. 6:**
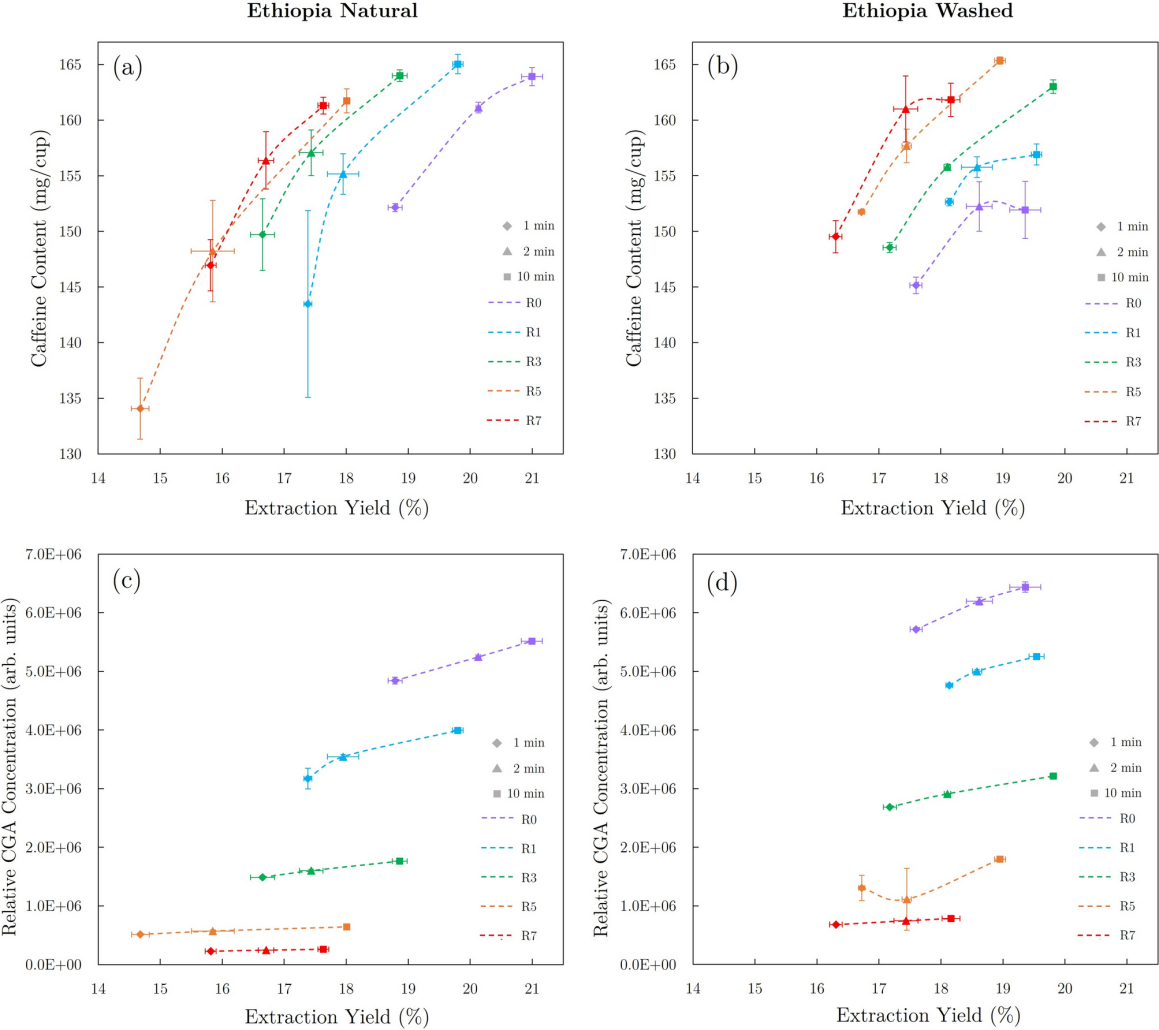
Plots of (**a**,**b**) caffeine concentration and (**c**,**d**) relative CGA concentration (5-CQA) vs extraction yield in natural (left) and washed (right) Ethiopian coffee brews for all investigated degrees of roast and brew times. Spline fits were used to clearly differentiate data sets for different degrees of roast.

As shown in Fig. 5, the degree of roast significantly affected CGA concentrations in brewed coffee samples, while the impact on caffeine content was evident but less pronounced. Respectively, caffeine concentrations ranged from 134–165 and 145–165 mg per (8 oz) cup in natural and washed Ethiopia coffee brews. The more limited ranges of extraction yields and caffeine concentrations for the washed Ethiopia samples are apparent when compared to the same data set for the natural Ethiopian samples in Fig. 6. This disparity demonstrates that the generally darker roast degrees achieved for the natural Ethiopia samples resulted in decreased minimum and average values of caffeine concentration and extraction yield (relative to common roast designations for the washed Ethiopian coffee).

For a common value of extraction yield, results shown in Fig. 6a,b indicate that caffeine levels show an increasing trend with the degree of roast if brews produced from darker roast batches can attain the same extraction yield as brews from lighter roast batches. However, since darker roasts typically resulted in lower overall extraction yields, caffeine concentrations were generally observed to decrease for darker roast batches. As the degree of roast approached extremely light levels, caffeine concentrations were also generally observed to decrease, likely due to inhibited extraction kinetics caused by decreased porosities of roasted seeds at lighter degrees of roast.

#### CGA content

In agreement with results from previous studies^9,23,24,28,29,53,54^, CGA concentrations in brewed samples showed drastic decreases as the degree of roast was increased. While increasing brew times and extraction yields resulted in higher CGA concentrations for a common roast batch, degree of roast was the dominant variable determining CGA content for all samples. In contrast to the extraction yield dependence observed for caffeine, CGA concentrations decreased with increasing degree of roast for all reported extraction yields (see Fig. 6c,d). Similar to caffeine results, the darker roast degrees achieved for the natural Ethiopia samples resulted in decreased CGA concentrations relative to washed Ethiopian samples with the same roast batch designations.

**Fig. 7:**
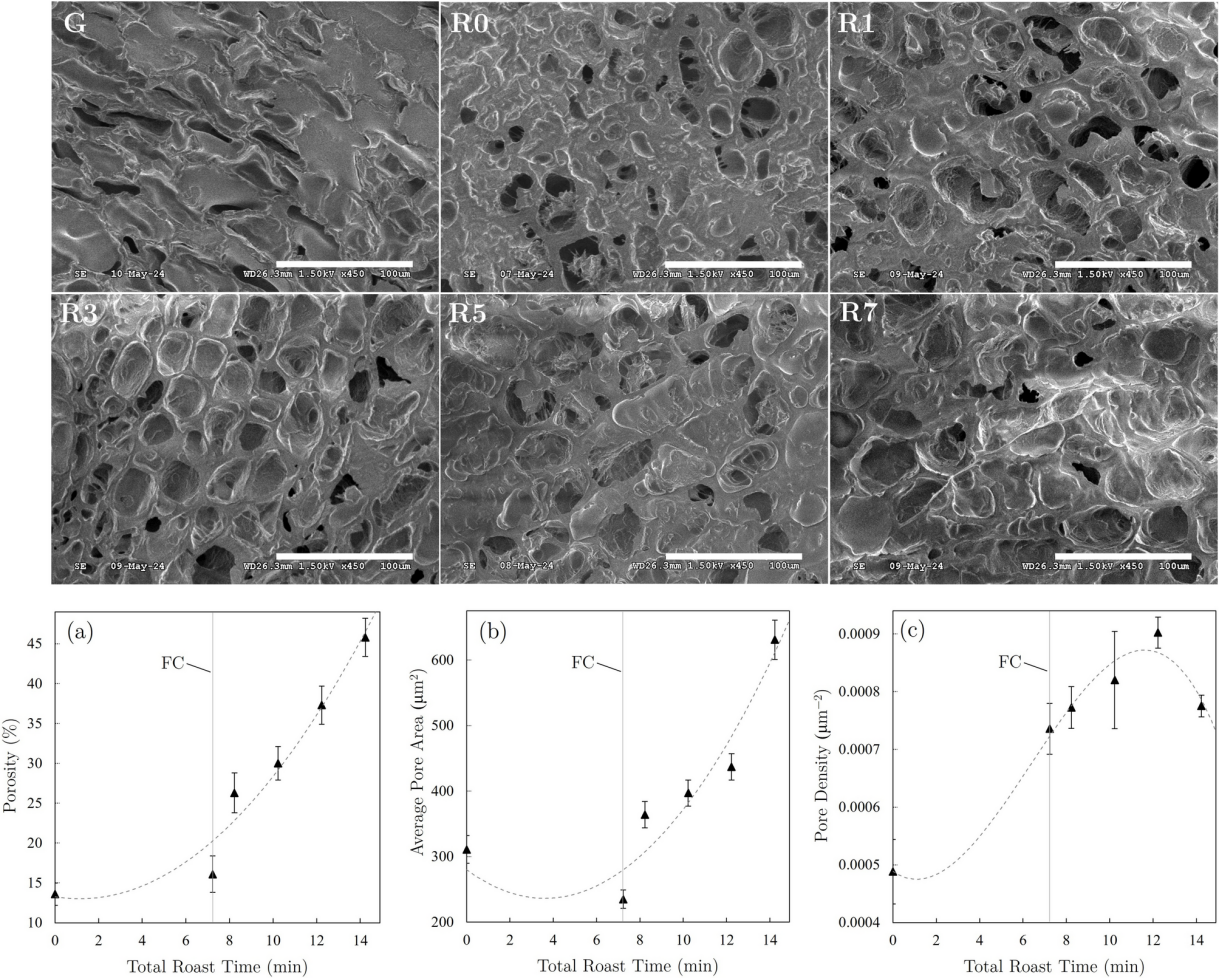
SEM images showing porosity evolution from green coffee (G) through R0$\rightarrow$R7 roast batches of washed Ethiopian coffee (scale bar = 100 $\upmu$m), and plots of (**a**) fractional porosity, (**b**) average pore area, and (**c**) pore density of the washed Ethiopia coffee as a function of total roast time (FC times are indicated by vertical lines).

#### Porosity

Scanning electron microscopy images shown in Fig. 7 depict visual changes in porosity as a function of increasing degree of roast and average values of fractional porosity, pore area, and pore density for the washed Ethiopian coffee are depicted as a function of roast time. While the elliptical mapping method does not provide 3D information related to the open or closed nature of the imaged pores, it does offer a 2D approximation of changes in porosity as a function of degree of roast. Fractional porosity measurements ranged from 13.6% for the unroasted green coffee to 45.8% for the R7 roast batch, and a quadratic fit applied to the data shows general agreement with previous results^55,56^.

Figure 7b shows an unexpected decrease in average pore area of the R0 roast batch relative to the unroasted green coffee. This is likely due to changes in pore shape during roasting that cause many of the elongated, individual pores within the green coffee to split into 2 or more pores as the seeds expel moisture and expand. Average pore areas measured in subsequent degrees of roast followed a roughly quadratic trend. Pore densities showed a general increase for all degrees of roast until a decrease was observed for the R7 roast batch. This decrease is due to significant increases in average pore area with extended roast times, which results in fewer pores over the same imaging region.

SEM images offering additional visual comparisons of coffee seed porosity as a function of degree of roast are displayed in the Supplementary Materials and clearly demonstrate that porosity increases tend to initiate in the central portion of the embryonic layer of the seed. Porosity increases were observed to progress from this central region within the endosperm towards the seed surface as the degree of roast increased.

## Discussion

This study demonstrates that degree of roast plays a significant role in resulting porosity and extracted compound concentrations in roasted coffee. While CGA concentrations drastically decreased with increasing degree of roast (which was an expected result due to thermal degradation during roasting^57^), changes in caffeine concentrations were more subtle and complex. Sublimation of caffeine at atmospheric pressure occurs at 352 $^{\circ }$F (178 $^{\circ }$C)^58^. Considering that all roasts performed in this study exceeded this temperature for some portion of the roast, some volatilization of caffeine via sublimation is an expected result^59^. However, the temperature of the inner volume of the coffee seeds is systematically lower than that measured by the bean probe for the majority of the roasting process^34^. Additionally, the actual sublimation temperature for caffeine under roasting conditions is likely higher than the quoted literature value due to elevated pressures within the seeds during roasting^33,60^.

**Fig. 8:**
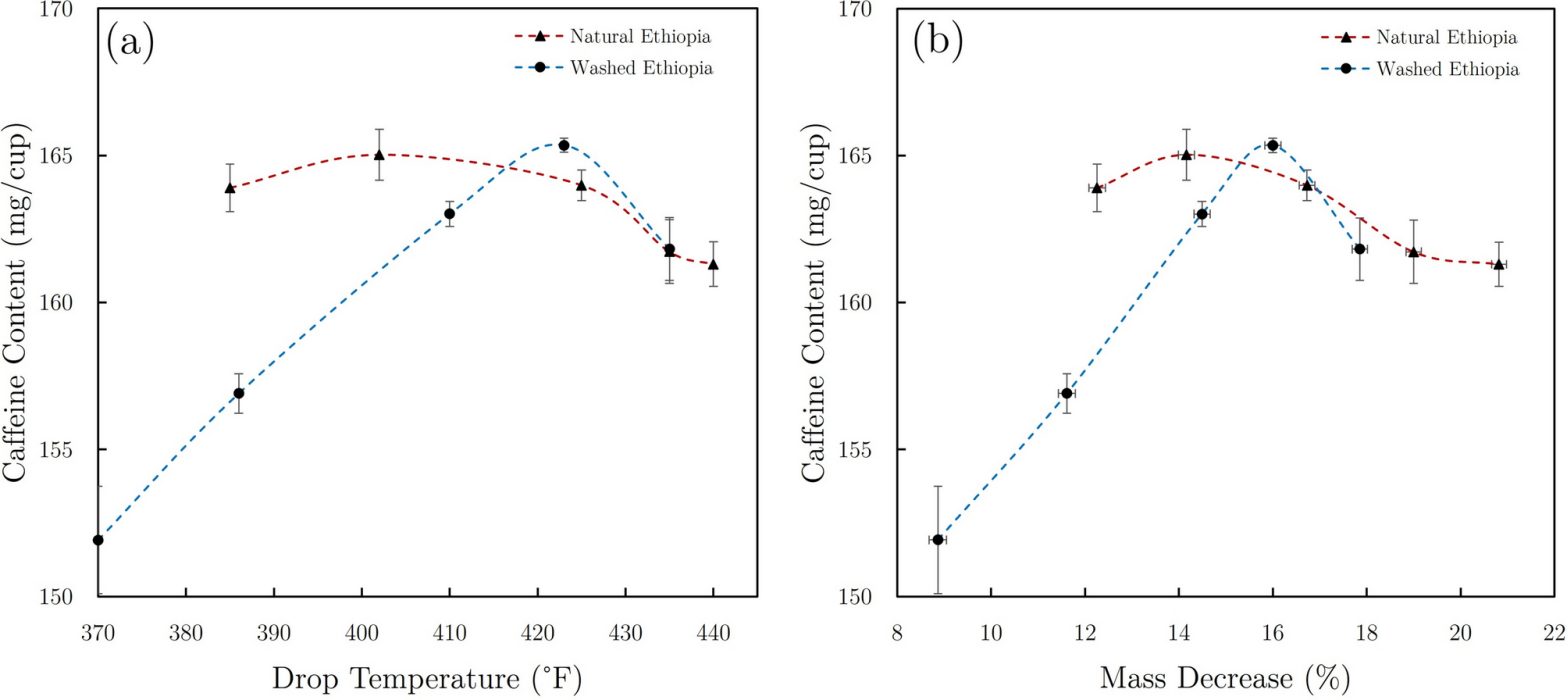
Plots of (**a**) caffeine content vs drop temperature and (**b**) caffeine content vs mass decrease for 10-min brews of natural and washed Ethiopian coffees. Spline fits were used to clearly differentiate data sets for each coffee variety.

Extracted caffeine in 10-min brews initially increased for roast batches that were terminated at temperatures above the nominal sublimation temperature, but caffeine content eventually reached a maximum value and began decreasing with increasing degree of roast (see Fig. 8a). This result is likely due to combined effects from higher efficiency extraction kinetics (attributed to increasing porosity), as well as caffeine losses due to sublimation during the later stages of roasting. Maximum caffeine levels were achieved at lower values of roasting loss and drop temperature for the natural coffee than the washed coffee, likely due to the systematically darker degrees of roast attributed to the natural Ethiopian roast batches. When caffeine content is plotted versus mass loss during roasting for both the natural and washed coffees (see Fig. 8b), caffeine levels reached a maximum for batches with roasting losses of approximately 14–16% and decreased for higher values. This result suggests that caffeine did indeed leave the coffee seeds during roasting after a certain degree of roast was achieved, but Fig. 8a demonstrates that sublimation effects did not become significant until the bean probe temperature exceeded values of approximately 400–420 $^{\circ }$F. Overall, extraction yield seems to play a more impactful role on resulting caffeine content in brewed coffee than the degree of roast alone.

Extraction yields were generally observed to decrease with increasing degree of roast for roasting mass losses greater than approximately 12–14%. Darker roasts showed a tendency to require lower extraction yields to achieve identical caffeine concentrations as lighter roasted coffees. This is likely due to higher relative concentrations of other compounds (that contribute to TDS and extraction yield measurements) present in lighter roasts that either decompose or volatilize as roasting progresses. However, decreased extraction yields observed for darker roasts resulted in lower caffeine concentrations than light and medium roasts (under identical grind and brew parameters). Since the range of extraction yields was constrained by a finite selection of brew times and grind distributions, no universal conclusion can be made for all possible degrees of roast and extraction yields. However, data collected in this study show a clear relationship between degree of roast, extraction yield, and caffeine content.

Porosity of the roasted coffee increased with the degree of roast and likely affected the sublimation rate of caffeine during roasting and extraction kinetics during brewing. The initial increase in extracted caffeine shown in Fig. 8 can be attributed to increasing porosity of roasted seeds, which creates additional pathways, pores, and surface area to facilitate more efficient diffusion processes during brewing. Extraction yields and caffeine concentrations eventually begin to decrease as the increasing degree of roast results in critical losses of remaining extractable compounds.

## Conclusions

While each green coffee variety exhibited slightly different behaviors relating extraction yield, caffeine content, and degree of roast, there were some common trends observed among the 90 total brew samples produced and analyzed in this study. Extraction yields showed maximum values for roast batches with $\sim$12–14% roasting mass loss (generally implying lighter roast levels) and decreased for all darker roast batches. Maximum values for caffeine concentrations (in 10-min brews) were observed in roast batches with $\sim$14–16% roasting losses (generally light to medium roast levels) and then decreased for all darker roast batches. This common behavior observed for both green coffee varieties roasted to a wide range of roast degrees provides indirect but convincing support for volatilization of caffeine via sublimation in the late stages of roasting. These results also suggest that the sublimation temperature of caffeine during roasting is likely higher than the documented 352 $^{\circ }$F (178 $^{\circ }$C) at atmospheric pressure.

Interestingly, if a common extraction yield can be achieved in brews of lighter and darker roasted coffees, results indicate that caffeine levels typically increase with the degree of roast. While this trend seems contradictory to observations of decreasing caffeine concentrations for darker roast batches, it is balanced by the generally lower extraction yields observed in brews made from darker roasted coffees. While the amount of caffeine may be lower in darker roasts, it nevertheless makes up a greater fraction of the remaining extractable material. Conversely, while higher caffeine concentrations may be present within the roasted seeds of extremely light degrees of roast, caffeine extraction in resulting brews is likely inhibited due to lower porosities attributed to lighter roast batches.

In general, extraction of caffeine and other compounds in coffee is primarily a function of two competing mechanisms: (1) porosity achieved through roasting that increases efficiency of extraction, and (2) volatilization and/or breakdown of compounds due to the roasting process. While this work demonstrates a clear relationship between extraction yield, porosity, caffeine content, and the degree of roast, additional research needs to be performed to determine whether these results extend to other roast, grind, and brew methods and parameters.

## Electronic supplementary material

Below is the link to the electronic supplementary material.

Supplementary Material 1

## Data Availability

The data that supports the findings of this study are available within the article and in the Supplementary Materials. However, additional data support and/or any desired figures with temperatures displayed in degrees Celsius ($^{\circ }C$) are available from the corresponding author upon reasonable request.
