# Supplementary material for: Caffeine content in filter coffee brews as a function of degree of roast and extraction yield
Source: Sci Rep. 2024 Nov 25;14:29126. doi: 10.1038/s41598-024-80385-3 (PMC11586412; doi:10.1038/s41598-024-80385-3)
Supplement: Supplementary file 1 — Supplementary Material 1 [file 41598_2024_80385_MOESM1_ESM.pdf]

Supplementary Materials for

"Caffeine Content in Filter Coffee Brews as a Function of Degree of Roast and Extraction Yield"

Scientific Reports Submission

Zachary R. Lindsey[1,*]

Joshua R. Williams[2]

James S. Burgess[1]

Nathan T. Moore[1]

Pierce M. Splichal[1]

1. Berry College, Rome, GA, USA 31049;
2. Drexel University, Philadelphia, PA, USA 19104;

∗ Corresponding author; e-mail: zlindsey@berry.edu.

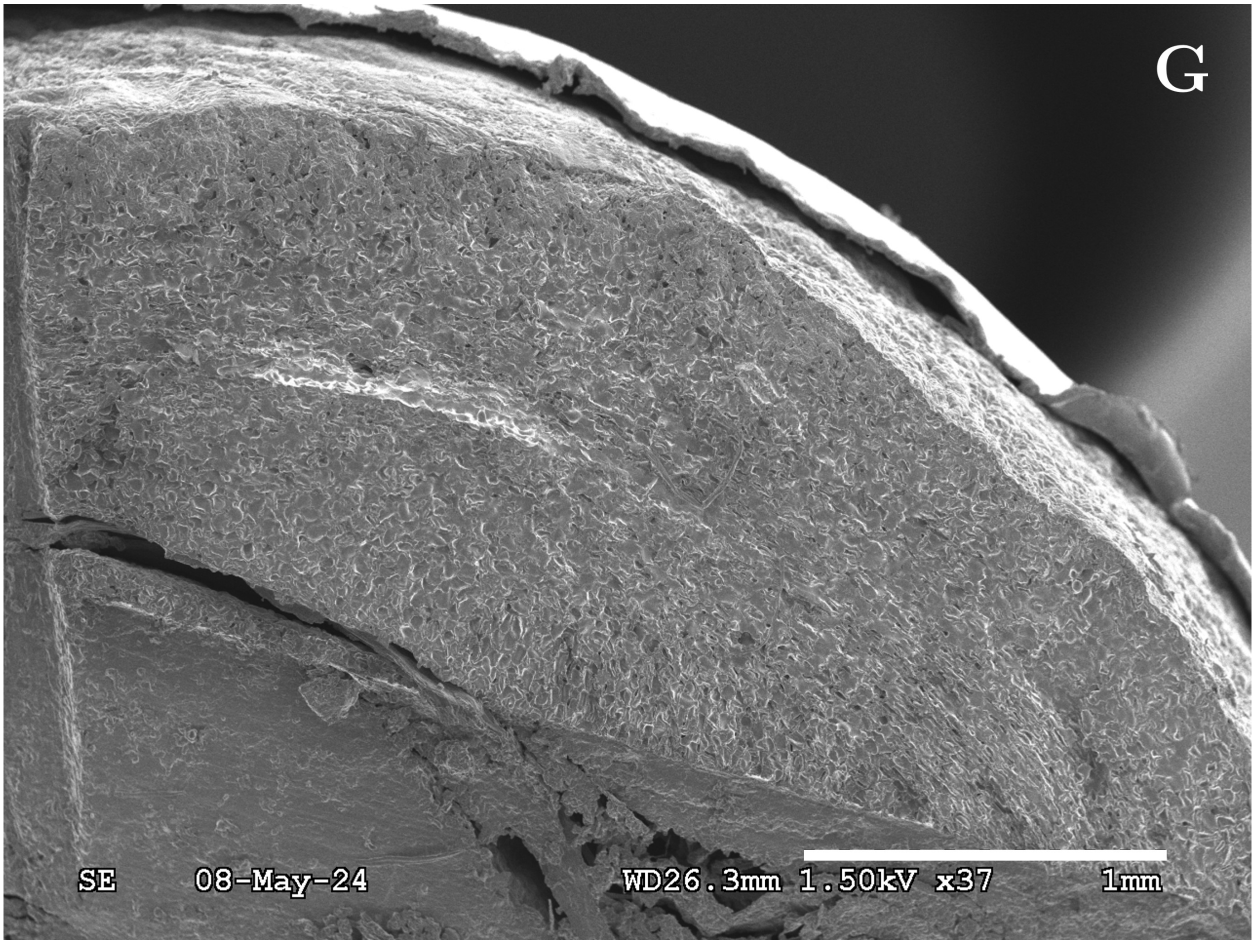

Figure S1: SEM image of a central cross section of a green (unroasted) seed of the washed Ethiopian coffee (scale bar = 1 mm).

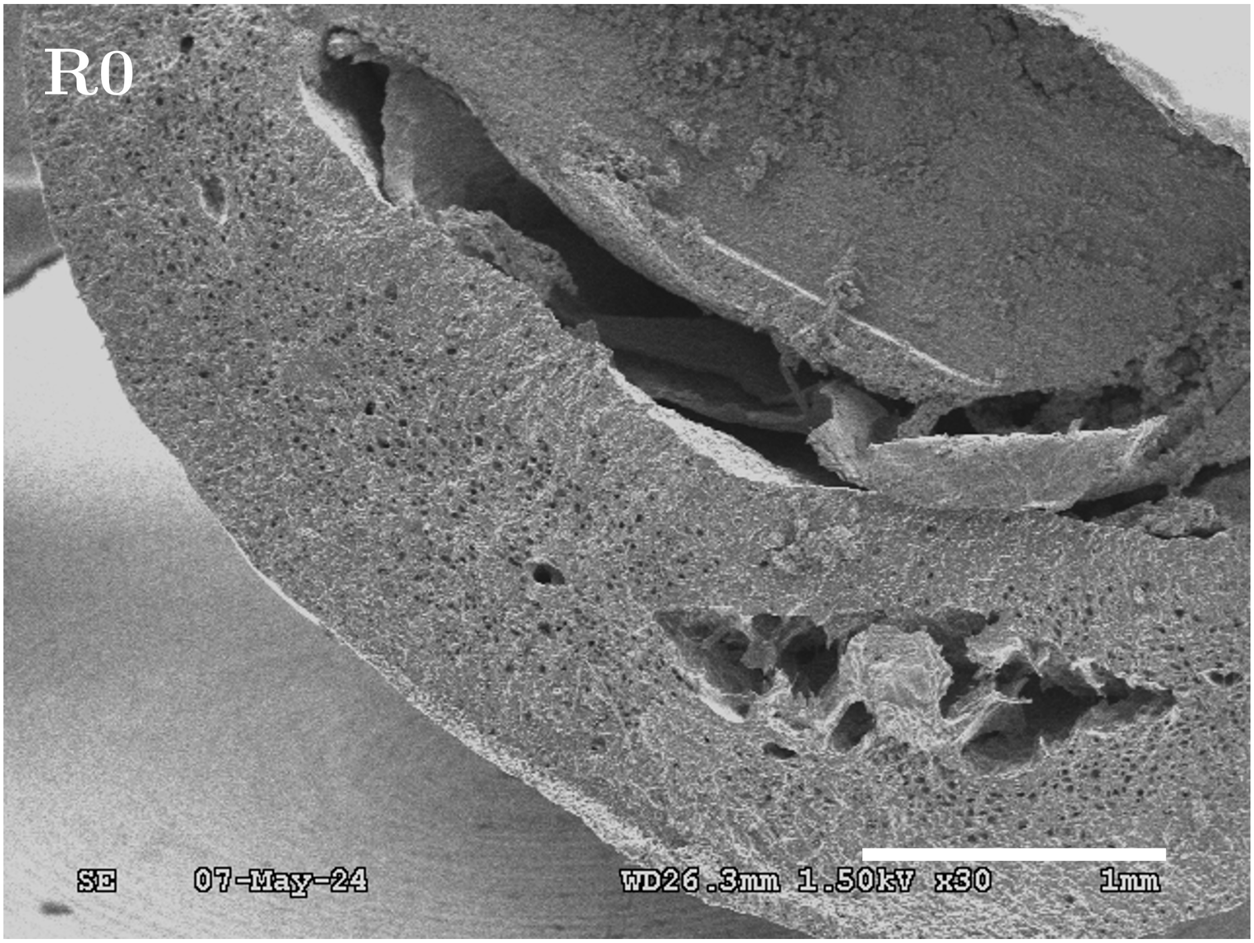

Figure S2: SEM image of a central cross section of a roasted seed from the R0 batch of the washed Ethiopian coffee (scale bar = 1 mm).

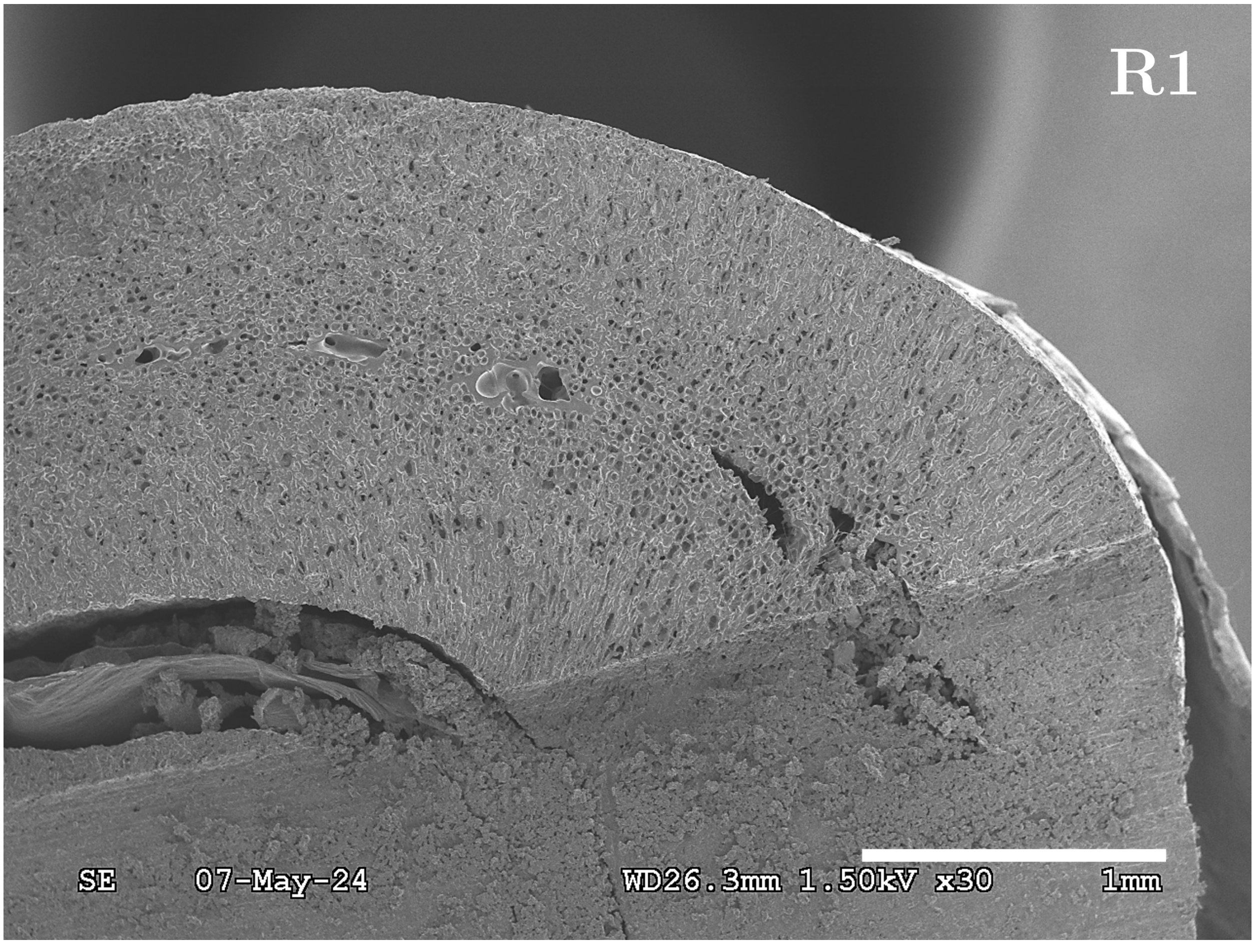

Figure S3: SEM image of a central cross section of a roasted seed from the R1 batch of the washed Ethiopian coffee (scale bar = 1 mm).

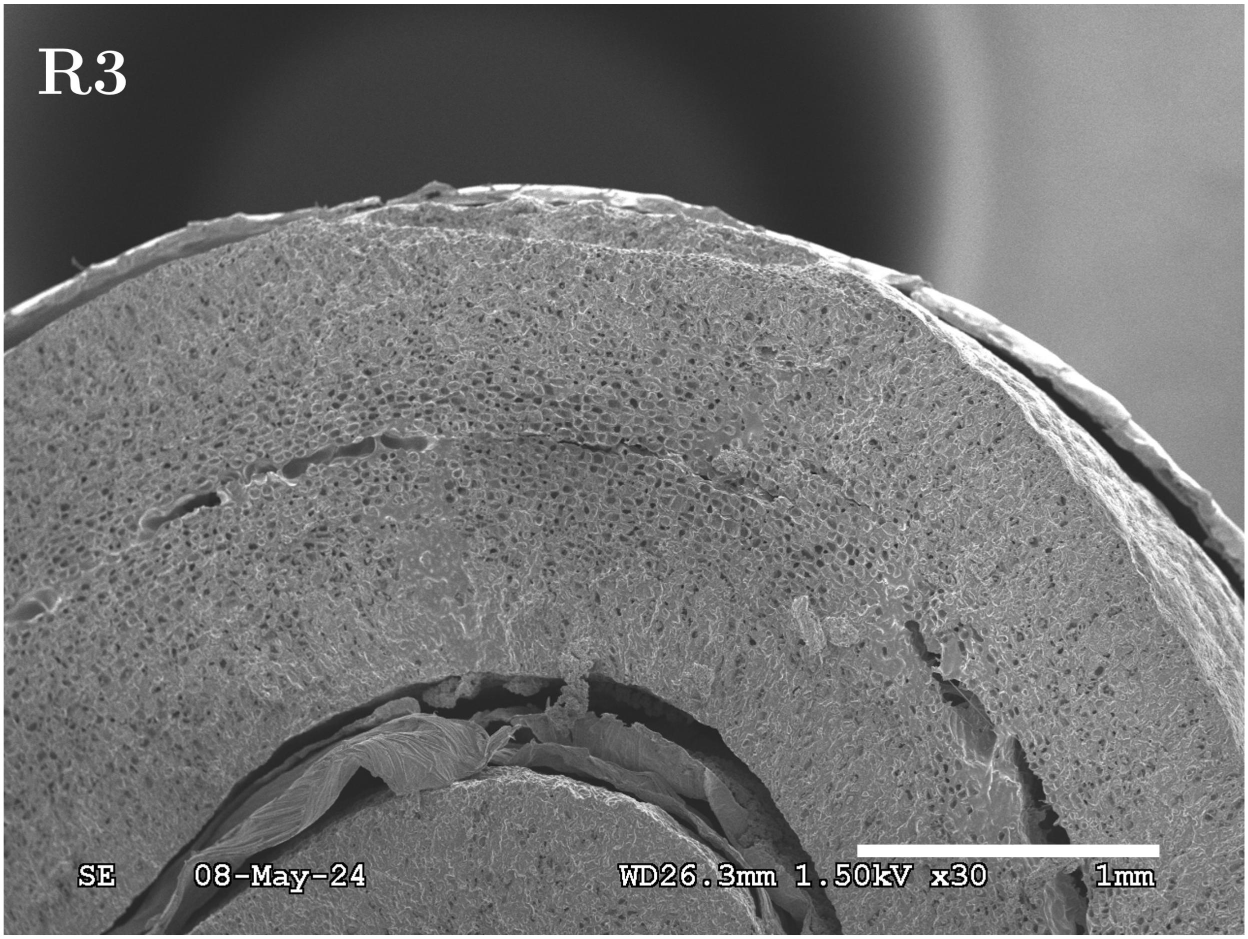

Figure S4: SEM image of a central cross section of a roasted seed from the R3 batch of the washed Ethiopian coffee (scale bar = 1 mm).

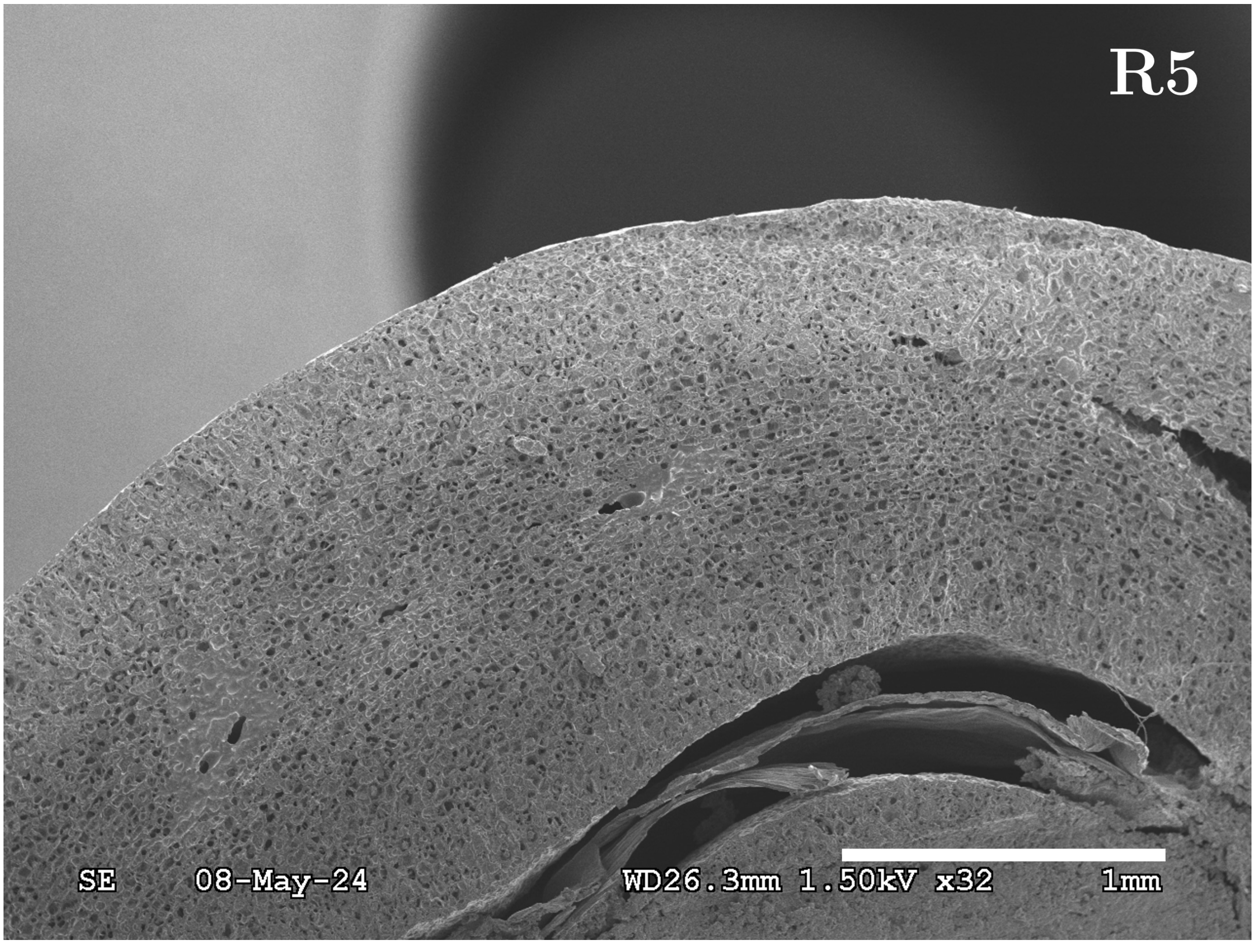

Figure S5: SEM image of a central cross section of a roasted seed from the R5 batch of the washed Ethiopian coffee (scale bar = 1 mm).

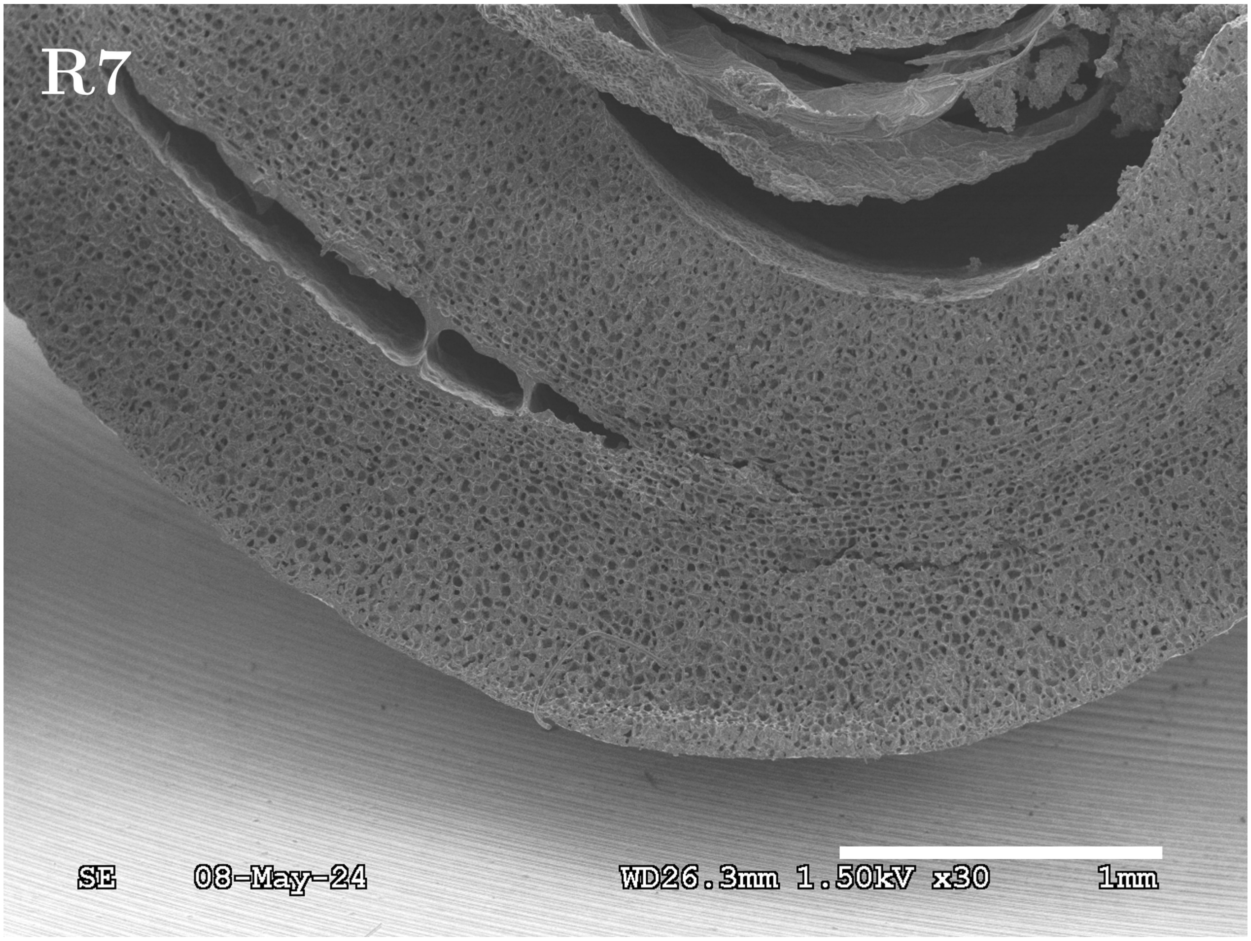

Figure S6: SEM image of a central cross section of a roasted seed from the R7 batch of the washed Ethiopian coffee (scale bar = 1 mm).

Table S1: Roast characterization data obtained for **natural** Ethiopian roast batches, including roasting mass loss (measured immediately post-roast), total roast time, drop temperature (T_{drop}), freely settled density, and Agtron color (reflectance) for whole (W) and ground (G) samples.

Batch	Mass Loss (%)	Roast Time (min)	T_{drop} (°F)	Density (g/mL)	Agtron Color (W/G)
R0	12.22	7.23	385	0.415	67 / 95
R1	14.02	8.25	402	0.359	54 / 73
R3	16.32	10.30	425	0.312	40 / 50
R5	18.28	12.30	435	0.280	31 / 36
R7	20.02	14.28	440	0.242	23 / 30

Table S2: Roast characterization data obtained for **washed** Ethiopian roast batches, including roasting mass loss (measured immediately post-roast), total roast time, drop temperature (T_{drop}), freely settled density, and Agtron color (reflectance) for whole (W) and ground (G) samples.

Batch	Mass Loss (%)	Roast Time (min)	T_{drop} (°F)	Density (g/mL)	Agtron Color (W/G)
R0	8.87	7.58	370	0.512	63 / 85
R1	11.50	8.55	386	0.423	56 / 69
R3	14.16	10.32	410	0.374	45 / 49
R5	15.48	12.36	423	0.356	40 / 43
R7	17.08	14.32	435	0.325	33 / 33

Table S3: Data obtained for **natural** Ethiopian coffee brews in terms of roast batch designation, roasting mass loss, brew time, total dissolved solids (TDS), extraction yield (EY), and caffeine concentration. Mass loss values obtained immediately post-roast are reported along with corresponding values measured after the 10-day degas period (in parentheses). Measurements for TDS, EY, and caffeine concentration were performed in triplicate, and mean values are reported along with corresponding standard errors.

Roast Batch	Mass Loss (%)	Brew Time (min)	TDS (%)	EY (%)	Caffeine (mg/cup)
R0	12.22 (12.26)	1	1.38 ± 0.02	18.8 ± 0.2	152.1 ± 0.4
		2	1.47 ± 0.01	20.1 ± 0.1	161.1 ± 0.5
		10	1.55 ± 0.02	21.0 ± 0.2	163.9 ± 0.8
R1	14.02 (14.16)	1	1.28 ± 0.01	17.4 ± 0.1	143.5 ± 8.4
		2	1.31 ± 0.02	18.0 ± 0.3	155.2 ± 1.8
		10	1.47 ± 0.01	19.8 ± 0.1	165.0 ± 0.9
R3	16.32 (16.73)	1	1.23 ± 0.02	16.7 ± 0.2	149.7 ± 3.2
		2	1.29 ± 0.01	17.4 ± 0.2	157.1 ± 2.1
		10	1.41 ± 0.01	18.9 ± 0.1	164.0 ± 0.5
R5	18.28 (19.00)	1	1.07 ± 0.02	14.7 ± 0.1	134.1 ± 2.7
		2	1.18 ± 0.03	15.9 ± 0.3	148.2 ± 4.6
		10	1.33 ± 0.01	18.0 ± 0.1	161.7 ± 1.1
R7	20.02 (20.82)	1	1.15 ± 0.01	15.8 ± 0.1	147.0 ± 2.3
		2	1.23 ± 0.01	16.7 ± 0.1	156.4 ± 2.6
		10	1.32 ± 0.01	17.6 ± 0.1	161.3 ± 0.8

Table S4: Data obtained for **washed** Ethiopian coffee brews in terms of roast batch designation, roasting mass loss, brew time, total dissolved solids (TDS), extraction yield (EY), and caffeine concentration. Mass loss values obtained immediately post-roast are reported along with corresponding values measured after the 10-day degas period (in parentheses). Measurements for TDS, EY, and caffeine concentration were performed in triplicate, and mean values are reported along with corresponding standard errors.

Roast Batch	Mass Loss (%)	Brew Time (min)	TDS (%)	EY (%)	Caffeine (mg/cup)
R0	8.87 (8.88)	1	1.28 ± 0.01	17.6 ± 0.1	145.2 ± 0.5
		2	1.37 ± 0.01	18.6 ± 0.2	152.2 ± 1.6
		10	1.42 ± 0.02	19.4 ± 0.3	151.9 ± 1.8
R1	11.50 (11.62)	1	1.32 ± 0.01	18.1 ± 0.1	152.6 ± 0.3
		2	1.36 ± 0.01	18.6 ± 0.1	155.8 ± 0.7
		10	1.44 ± 0.01	19.5 ± 0.1	156.9 ± 0.7
R3	14.16 (14.49)	1	1.24 ± 0.01	17.2 ± 0.1	148.5 ± 0.3
		2	1.32 ± 0.01	18.1 ± 0.1	155.8 ± 0.2
		10	1.44 ± 0.01	19.8 ± 0.1	163.0 ± 0.4
R5	15.48 (16.00)	1	1.21 ± 0.01	16.7 ± 0.1	151.8 ± 0.1
		2	1.27 ± 0.01	17.5 ± 0.1	157.7 ± 1.1
		10	1.39 ± 0.01	19.0 ± 0.1	165.4 ± 0.2
R7	17.08 (17.85)	1	1.17 ± 0.01	16.3 ± 0.1	149.5 ± 1.0
		2	1.26 ± 0.01	17.4 ± 0.2	161.0 ± 2.1
		10	1.34 ± 0.01	18.2 ± 0.2	161.8 ± 1.1

Table S5: Roast data obtained for **natural** Ethiopian coffee roast batches regarding measured first crack (FC) event times, as well as absolute and relative roast times spent in the drying, middle, and post-FC development phases. Absolute times are reported for each phase with corresponding relative percentages in parentheses.

Roast Batch	Drying Phase (min)	Middle Phase (min)	Development Phase (min)	FC (min)
R0	3.65 (50.8%)	3.53 (49.2%)	0.00 (0.0%)	7.18
R1	3.73 (45.5%)	3.48 (42.4%)	1.00 (12.1%)	7.22
R3	3.70 (36.0%)	3.57 (34.8%)	3.00 (29.2%)	7.27
R5	3.75 (30.6%)	3.50 (28.6%)	5.00 (40.8%)	7.27
R7	3.75 (26.3%)	3.50 (24.6%)	7.00 (49.1%)	7.25

Table S6: Roast data obtained for **washed** Ethiopian coffee roast batches regarding measured first crack (FC) event times, as well as absolute and relative roast times spent in the drying, middle, and post-FC development phases. Absolute times are reported for each phase with corresponding relative percentages in parentheses.

Roast Batch	Drying Phase (min)	Middle Phase (min)	Development Phase (min)	FC (min)
R0	4.10 (54.3%)	3.45 (45.7%)	0.00 (0.0%)	7.55
R1	4.12 (48.2%)	3.42 (40.1%)	1.00 (11.7%)	7.53
R3	4.08 (38.8%)	3.44 (32.7%)	3.00 (28.5%)	7.53
R5	4.08 (32.5%)	3.47 (27.7%)	5.00 (39.8%)	7.57
R7	4.00 (27.6%)	3.52 (24.2%)	7.00 (48.2%)	7.52

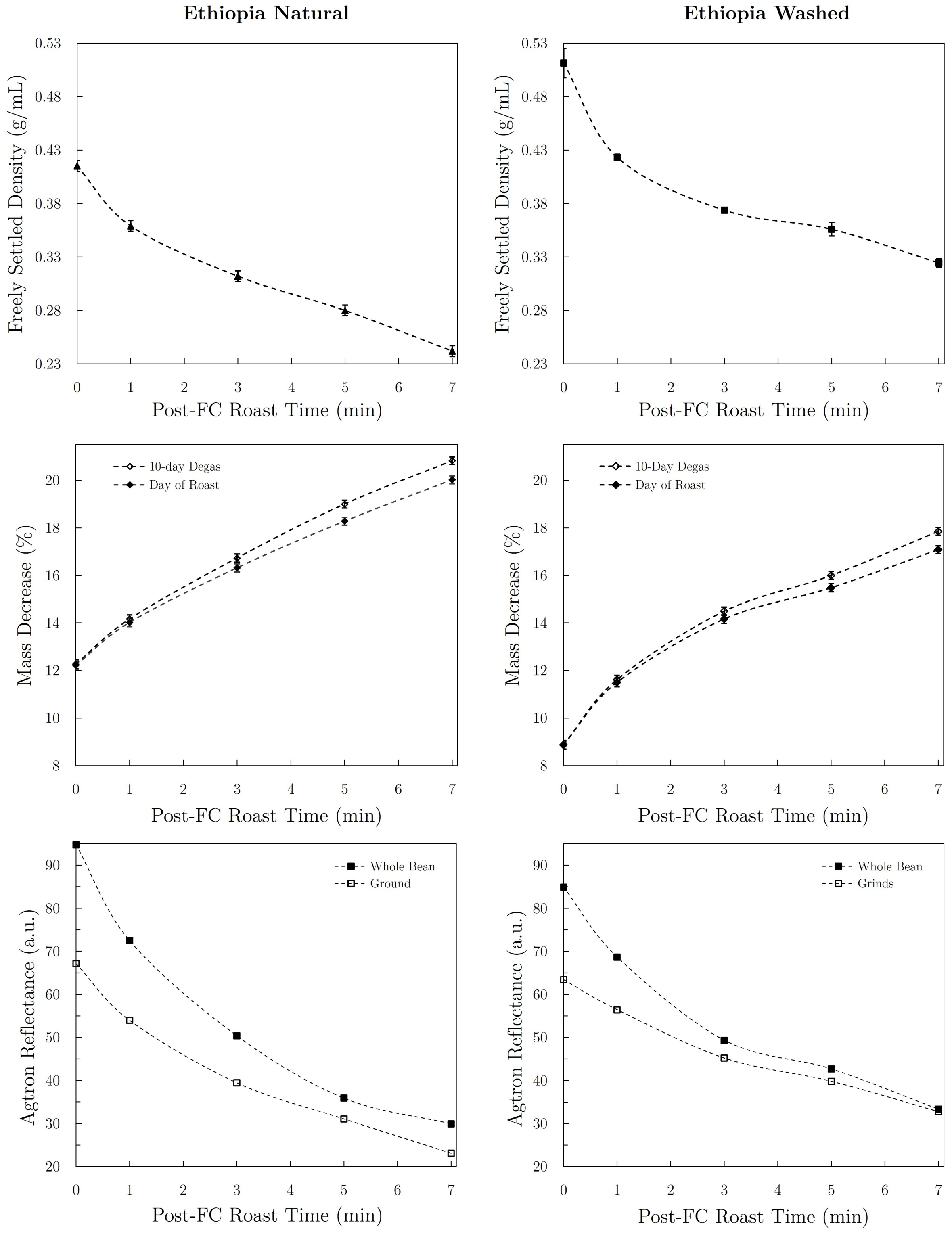

Figure S7: Plots of measured (a) density, (b) mass decrease, and (c) color/reflectance as a function of roast time after the onset of first crack (FC) for the natural and washed Ethiopian coffees.

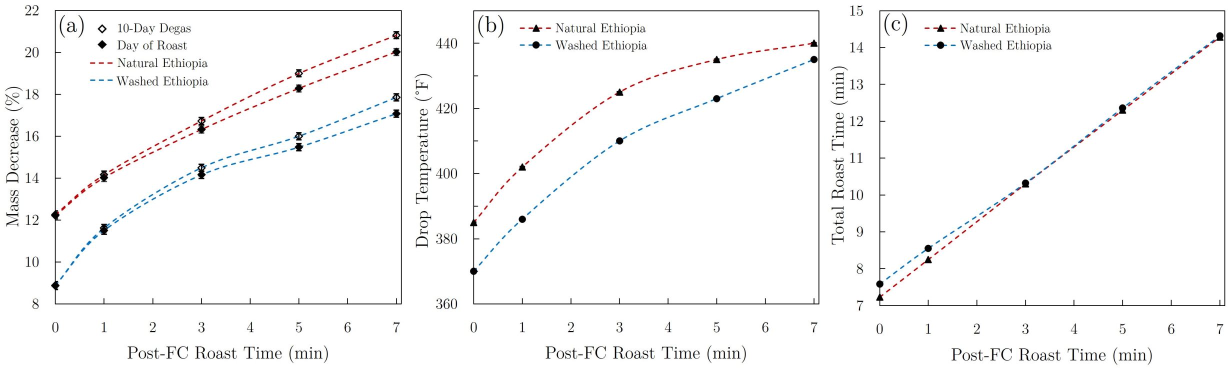

Figure S8: Plots of (a) mass decrease, (b) drop temperature, and (c) total roast time vs post-FC roast time for natural and washed Ethiopian coffees.
